# Effects of Web-Based Group Mindfulness Training on Stress and Sleep Quality in Singapore During the COVID-19 Pandemic: Retrospective Equivalence Analysis

**DOI:** 10.2196/21757

**Published:** 2021-03-15

**Authors:** Julian Lim, Zaven Leow, Jason Ong, Ly-Shan Pang, Eric Lim

**Affiliations:** 1 Centre for Sleep and Cognition Yong Loo Lin School of Medicine National University of Singapore Singapore Singapore; 2 Department of Neurology Center for Circadian and Sleep Medicine Northwestern University Feinberg School of Medicine Evanston, ID United States; 3 Brahm Centre Tampines Singapore

**Keywords:** mindfulness, COVID-19, videoconference, perceived stress, sleep quality, intervention, telehealth, mental health, psychology

## Abstract

**Background:**

The COVID-19 pandemic has negatively impacted psychological health. Mindfulness training, which helps individuals attend to the present moment with a nonjudgmental attitude, improves sleep and reduces stress during regular times. Mindfulness training may also be relevant to the mitigation of harmful health consequences during acute crises. However, certain restrictions may necessitate the web-based delivery of mindfulness training (ie, rather than in-person group training settings).

**Objective:**

The objective of our study was to examine the effects of mindfulness interventions during the COVID-19 pandemic and to evaluate the effectiveness of web-based interventions.

**Methods:**

Data from an ongoing study were used for this retrospective equivalence analysis. Recruited participants were enrollees from mindfulness courses at a local charity organization that promoted mental wellness. This study had no exclusion criteria. We created three groups; two groups received their training during the COVID-19 pandemic (in-person training group: n=36; videoconferencing group: n=38), and a second control group included participants who were trained before the pandemic (n=86). Our primary outcomes were self-reported stress and sleep quality. Baseline levels and changes in these variables due to mindfulness training were compared among the groups via an analysis of covariance test and two one-tailed *t* tests.

**Results:**

Baseline perceived stress (*P*=.50) and sleep quality (*P*=.22) did not differ significantly among the three groups. Mindfulness training significantly reduced stress in all three groups (*P*<.001), and this effect was statistically significant when comparing videoconferencing to in-person training (*P*=.002). Sleep quality improved significantly in the prepandemic training group (*P*<.001). However, sleep quality did not improve in the groups that received training during the pandemic. Participants reported that they required shorter times to initiate sleep following prepandemic mindfulness training (*P*<.001), but this was not true for those who received training during the pandemic. Course attendance was high and equivalent across the videoconferencing and comparison groups (*P*=.02), and participants in the videoconferencing group engaged in marginally more daily practice than the in-person training group.

**Conclusions:**

Web-based mindfulness training via videoconferencing may be a useful intervention for reducing stress during times when traditional, in-person training is not feasible. However, it may not be useful for improving sleep quality.

## Introduction

During times of crisis, it is important that individuals are equipped with tools for coping with the psychological impacts of change and uncertainty. In this retrospective study, we investigated the effects of mindfulness training in terms of reducing stress and improving sleep quality during the COVID-19 pandemic, which is an event that has resulted in major economic and social disruptions worldwide. More than 7 million confirmed SARS-CoV-2 infections and 400,000 deaths resulting from COVID-19 have been reported as of June 10, 2020.

Studies that have been conducted during the COVID-19 pandemic have reported increases in the incidence of depression, anxiety, and stress across diverse populations [1]. Altered sleep habits and circadian rhythm misalignment resulting from disruptions in routines (ie, those caused by quarantine and lockdowns) may further exacerbate these problems [2,3]. Cross-sectional data have suggested that social support may be a protective factor against the negative consequences of stress [4], and this has promoted the use of psychosocial interventions in stress-affected communities.

Mindfulness practice has been reported as an effective method for coping with stressors, as it provides individuals with flexible strategies for relating to thoughts and emotions. It has also been suggested that mindfulness practice is a potential intervention for stress reduction during the COVID-19 pandemic [5]. In psychiatric research, mindfulness is commonly defined as the awareness that arises from paying purposeful attention to the present moment experience in a nonjudgmental manner [6]. It is commonly taught and cultivated through standardized curricula, such as the Mindfulness-Based Stress Reduction program [7]. Considerable emphasis has been placed on the importance of having a mindful disposition in daily living and engaging in habitual mindfulness practice after the conclusion of formal training.

Meta-analyses have demonstrated that mindfulness has moderate effects in terms of reducing stress in healthy individuals [8] and the incidence of psychopathology [9]. This buffering effect has been observed in laboratory paradigms that acutely induce stress [10,11] and people who experience stress on a chronic basis. This reduction in stress may have beneficial health sequelae, such as decreasing the incidence of harmful behaviors (eg, smoking) [12] and reducing susceptible individuals’ likelihood of developing serious depressive or anxiety disorders (per diathesis-stress models) [13].

A related but separate body of research has highlighted the beneficial effects of mindfulness training on sleep quality. High dispositional mindfulness correlates with improved self-reported sleep quality across several populations [14], and a growing number of randomized controlled trials have shown improvements in sleep quality after mindfulness instruction in both nonclinical [15] and clinical populations [16]. Regular poor sleep is associated with the significant deterioration of short- and medium-term quality of life, and as with chronic stress, regular poor sleep may predispose individuals to developing serious psychological problems over time [17].

Delivering mindfulness training in traditional group settings may be challenging during a pandemic due to restrictions such as social distancing and lockdowns. Group videoconferencing is an attractive work-around method for providing mindfulness instruction and preserving the high-quality facilitation and social and communal aspects of mindfulness interventions. However, there have been few studies that investigate whether this mode of delivery is as effective as in-person instruction and whether good adherence to a mindfulness program (ie, attendance and daily practice) can be achieved via videoconferencing. There are also very little data on the effects of mindfulness interventions that are conducted during the COVID-19 pandemic. These knowledge gaps motivated us to conduct this study. Specifically, we hypothesized that mindfulness training delivered via videoconferencing would have an effect that is equivalent to that of in-person training in terms of reducing stress and improving sleep quality.

## Methods

### Aim and Hypotheses

**Figure 1:**
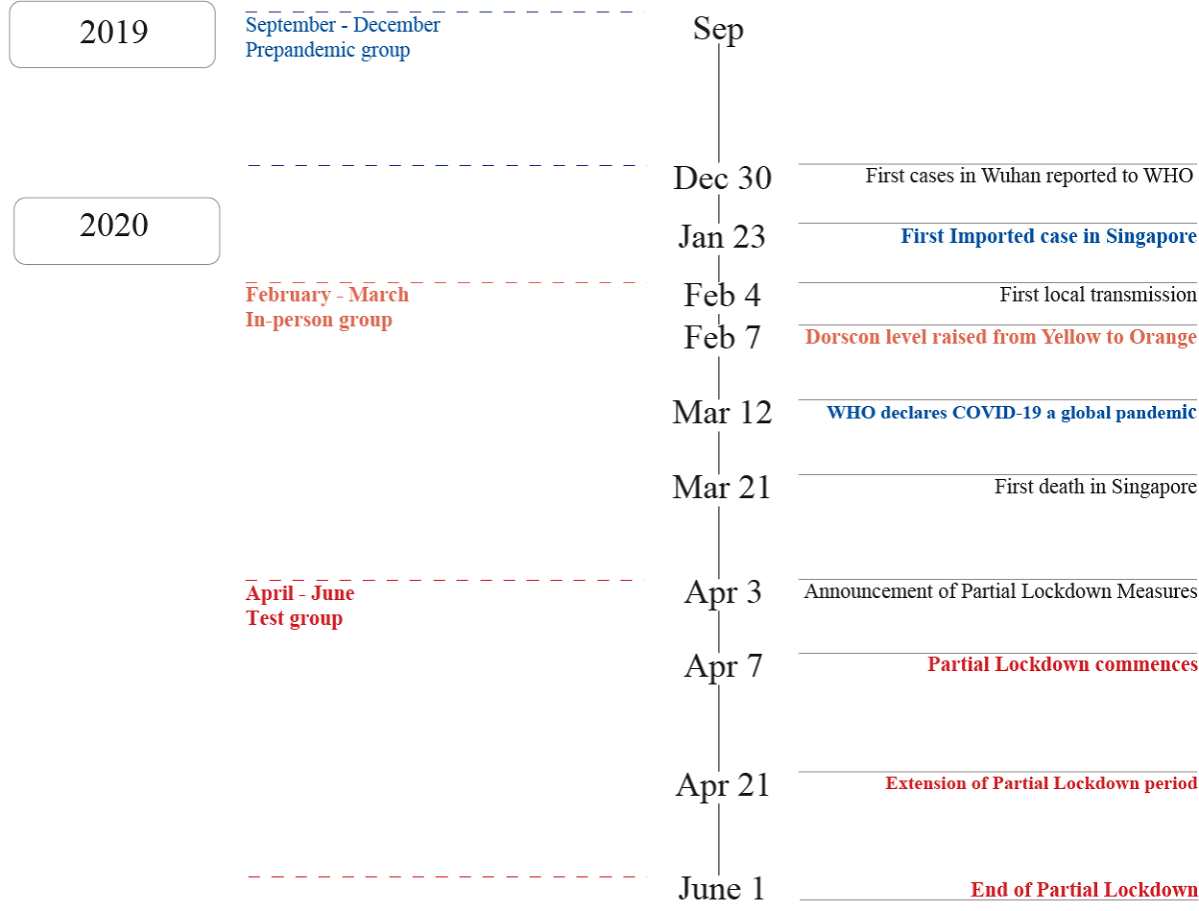
Timeline of key COVID events in Singapore and recruitment periods.

The primary aims of this study were to examine the effects that group mindfulness interventions have on stress and sleep quality during a global crisis (ie, the COVID-19 pandemic) and to determine whether the web-based delivery of mindfulness training was equivalent to traditional, in-person classes. Data from two comparison groups and a control group were analyzed. The first comparison group was composed of participants who underwent training during a period of heightened alert resulting from the community spread of SARS-CoV-2 (ie, February to March 2020), and the second comparison group was composed of participants who underwent mindfulness instruction via group videoconferencing classes that were led by an experienced facilitator during a period of partial lockdown (ie, April to May 2020) (Figure 1). We had 2 hypotheses. For our first hypothesis, we predicted that participants would experience higher levels of perceived stress and poorer sleep quality at baseline during the pandemic than before the pandemic (ie, the control period). For our second hypothesis, we tested whether web-based training (ie, during the lockdown period) was equivalent to in-person training (ie, before and during the COVID-19 pandemic), and we predicted that web-based training would be equivalent to in-person training (ie, before and during the pandemic) in terms of reducing stress and improving sleep quality.

### Setting

This study was conducted with people from the general community. During the prelockdown period, participants attended courses at one of the Brahm Centre sites [18], which were located in community hospitals or housing estates around Singapore. During the lockdown period, participants remotely attended mindfulness training from their homes. Questionnaires were completed and submitted via a web-based platform.

### Procedure

The data in this analysis were collected from an ongoing study that investigated the effects of baseline variables on mindfulness training. The participants in the ongoing study were enrollees from 1 of 3 different mindfulness courses that are offered by Brahm Centre, which is a charity organization that conducts wellness activities for the general community. The three courses were (1) the Mindfulness Foundation Course [15], (2) the Mindfulness Intermediate Course, and (3) Mindfulness-Based Stress Reduction [7]. Detailed descriptions of the courses that were offered in the ongoing study are reported in the Supplementary Information (Multimedia Appendix 1). The breakdown of the number of participants in each course is shown in Table S1. Although the courses differ in length (ie, 4 weeks for the foundation course and 8 weeks for the intermediate and Mindfulness-Based Stress Reduction courses), we noted that previous studies have generally not found evidence of a dose-response relationship between course length and psychological outcomes in mindfulness training [19,20].

Upon enrolling in one of these courses, participants were invited to take part in this study by completing a set of questionnaires via SurveyMonkey (SurveyMonkey Inc) [21]. These questionnaires included the Perceived Stress Scale (PSS) and the Pittsburgh Sleep Quality Inventory (PSQI). Data from other questionnaires in the survey packet are not discussed further in this paper. Participants were provided with study information and informed that they were providing implicit consent for their participation in this study by completing and submitting the questionnaires. They were required to complete the surveys within 24 hours of the first session of each course. The mindfulness courses consisted of 4 or 8 sessions that were taught by 1 of 2 instructors who were certified by the Centre For Mindfulness at the University of Massachusetts Medical School. These instructors had at least 1000 hours of teaching experience. Web-based classes were delivered via the Zoom videoconferencing platform (Zoom Video Communications Inc) [22]. Participants were strongly encouraged to practice mindfulness exercises (ie, the exercises they were taught) on a daily basis. Following the last session of each course, participants answered the items of the PSS and PSQI again and reported on their average daily mindfulness practice times over the duration of the course.

### Participants

**Figure 2:**
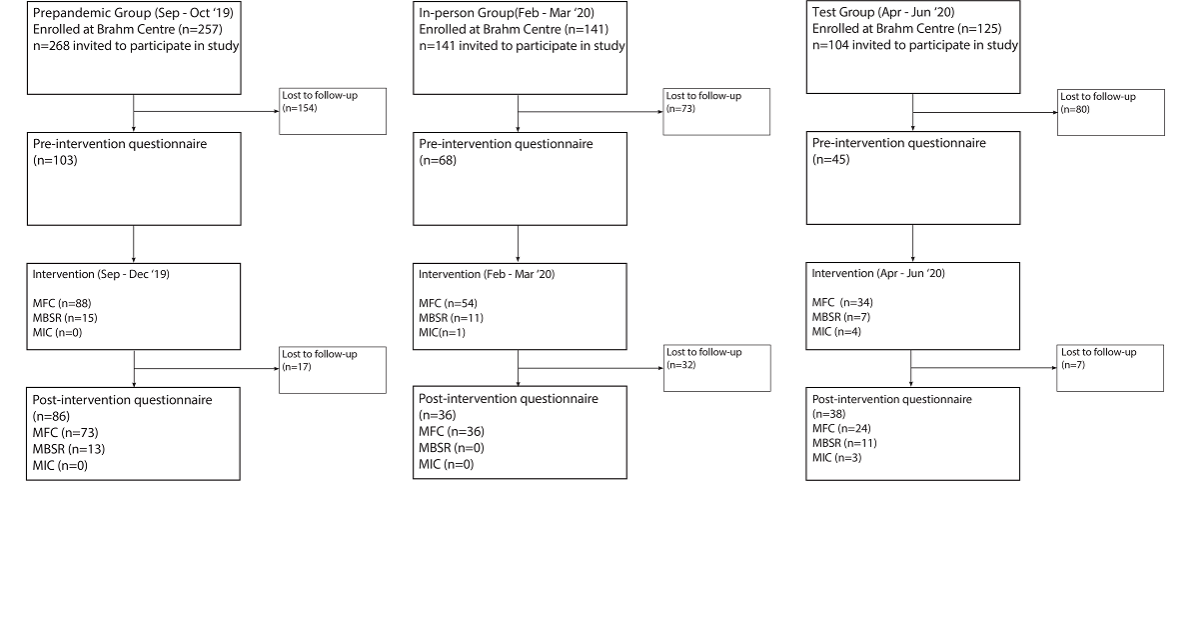
Diagram of participant flow throughout the protocol. Participants were enrolled in 1 of 3 types of mindfulness courses: 4-week Mindfulness Foundation Course (MFC), 8-week Mindfulness Based Stress Reduction (MBSR), and 4-week Mindfulness Intermediate Course (MIC).

We created three different groups based on the periods when participants underwent their mindfulness training. These periods were relative to milestone events that occurred in Singapore during the COVID-19 pandemic. Since group assignment was dependent on the imposition and lifting of restrictions that were not within our control, prospectively randomizing participants into groups was not possible in this study. The test group was comprised of participants who were recruited in April and May 2020 (n=38), which roughly encompassed the period in which Singapore entered a partial lockdown. Participants in this group were provided with web-based group training. The in-person training group included participants (n=36) who signed up for face-to-face group courses that took place during the months of February and March 2020, which approximately corresponded with the period of heightened alert in Singapore. With regard to the prepandemic training group, we used data that were collected from a group of 86 participants who took part in this study from October to December 2019 (ie, the period prior to the first reported case of COVID-19 in Singapore). Participants were community-dwelling individuals who voluntarily signed up for and participated in the mindfulness program, and this study had no exclusion criteria. Figure 1 shows the timeline of landmark events (ie, those related to the pandemic in Singapore) and the periods of participant recruitment. Table 1 shows the baseline demographic and clinical characteristics of the three groups, and Figure 2 shows a diagram of participant flow.

**Table 1:** Participants’ baseline characteristics.

Characteristics			Prepandemic training group		In-person training group		Test group		*P* value^a^
Sample size, n			86		36		38		N/A^b^
**Demographic characteristics**									
	Age (years), mean (SD)	45.46 (11.71)		47.14 (10.98)		49.77 (11.17)		.15	
	Male, n	26		11		11		.99	
	Length of education (years), mean (SD)	16.44 (2.63)		15.47 (2.81)		15.61 (2.91)		.19	
	Previous meditation experience, n	16		3		10		.14	
**Race, n**									
	Chinese	79		36		36		N/A	
	Malay	2		0		1		N/A	
	Indian	3		0		1		N/A	
	Other	2		0		0		N/A	
**Clinical variables, mean (SD)**									
	Perceived Stress Scale score	20.17 (6.67)		21.19 (8.07)		19.26 (6.84)		.50	
	Pittsburgh Sleep Quality Inventory score	6.29 (2.96)		5.58 (2.98)		6.82 (3.27)		.22	
	Total sleep time (minutes)	406.69 (55.57)		402.38 (49.69)		405.23 (68.98)		.93	
	Sleep onset latency (minutes)	23.64 (25.76)		17.12 (12.66)		27.09 (28.72)		.20	

^a^*P* values were derived from the appropriate statistical test (ie, analysis of variance/Chi-square test) for comparing all three groups.

^b^N/A: not applicable.

### Measures

Our primary outcomes of interest were the global scores of two questionnaires. Both of these questionnaires are considered gold-standard instruments for measuring their respective constructs, as they have excellent psychometric properties.

#### PSS Instrument

The PSS [23] is a widely used 10-item instrument for measuring subjectively experienced stress.

#### PSQI Scale

The PSQI [24] is a 19-item scale that is commonly used to self-assess sleep quality and disturbances over a 1-month period. This questionnaire requires participants to report on their average sleep and wake times and the average time they take to fall asleep at night. Global PSQI scores of >5 are indicative of clinical sleep disturbance.

In addition to obtaining global PSQI scores, we conducted an exploratory analysis on the two following self-reported sleep variables: total sleep time and sleep onset latency (ie, the time taken to fall asleep).

### Statistical Analysis

We conducted a complete case analysis on participants who provided PSS and PSQI data both before and after the intervention. A one-way analysis of variance (ANOVA) test was conducted to identify differences in baseline PSS scores, baseline PSQI scores, and the amount of practice among the three groups. To compare the effects of the interventions that were delivered before and during the pandemic, we conducted a 2 × 3 repeated measures analysis of covariance (ANCOVA) test in which mindfulness training (ie, pretraining and posttraining) was used as a within-subjects factor and group type (ie, the prepandemic training, in-person training, and test groups) was used as a between-subjects factor. As sphericity assumptions were not violated, no corrections for nonsphericity were applied. Course duration (ie, 4 or 8 weeks) and participants’ previous meditation experience were coded as dummy variables and used as covariates. To establish equivalence between web-based and in-person training, we conducted two one-tailed *t* tests to compare the test group to the two control groups. We set the smallest effect size of interest (ie, Cohen *f*) to 0.29 for the comparison between the test group and the prepandemic training group and 0.38 for the comparison between the test group and the in-person training group. Per the recommendation of Lakens [25], the smallest effect size of interest was determined by computing the smallest effect size that our study could detect (thresholds: =.05; =.90). Statistical analysis was conducted with SPSS, version 23 for Mac (IBM Corporation).

### Ethical Approval

This study was approved by the National University of Singapore Institutional Review Board and conducted in accordance with the ethical standards of the 1964 Helsinki declaration and its later amendments. Participants were provided with an information sheet about the study. Participants provided implicit consent by completing and submitting the questionnaire. Identifying information was not collected, as per this study’s protocol.

### Data Sharing Statement

Deidentified participant data from this analysis will be freely available on the Open Science Framework website after publication [26].

## Results

### Demographics

Participants’ baseline, self-reported characteristics are presented in Table 1. The groups did not differ significantly in terms of age, gender, education level, or previous meditation experience.

### Perceived Stress

The one-way ANOVA test for the three groups showed that there were no significant differences in baseline levels of perceived stress (*F*_2,159_=0.69; *P*=.50). Baseline stress was within the moderate range (ie, a PSS score of 14-26). After comparing the effects of the interventions for each group, we found that training had a significant overall effect (*F*_1,155_=12.80; *P*<.001; partial ε^2^=.076). However, there were no interactions between the training and group factors (*F*_2,155_=0.92; *P*=.40) (Figure 2). Our planned posthoc comparisons showed that perceived stress significantly decreased in all three groups after mindfulness training (prepandemic training group: *t*_84_=7.55; *P*<.001; in-person training group: *t*_35_=2.58; *P*=.01; test group: *t*_37_=4.09; *P*<.001).

The two one-tailed *t* tests for comparing changes in PSS scores between the test group and the prepandemic training group (the smallest effect size of interest was set to 0.29) revealed that the observed effect size was significantly within the equivalence bounds (*t*_124_=2.10; *P*=.02). This indicated that web-based mindfulness training during the pandemic was equivalent to in-person training before the pandemic in terms of reducing stress. Similarly, the comparison between the test group and the in-person training group (the smallest effect size of interest was set to 0.38) indicated that web-based training during the pandemic was equivalent to in-person training during the pandemic (*t*_73_=−2.99; *P*=.002).

### Subjective Sleep Quality

**Figure 3:**
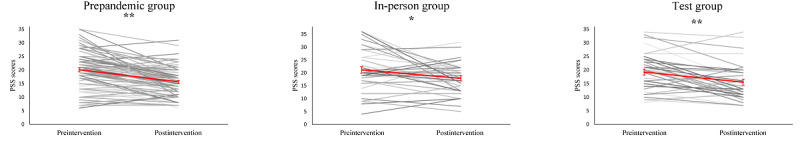
Change in perceived stress. Black lines depict change for individual participants, and red line indicates the mean change and standard errors in the group. Change in perceived stress from pre- to post-intervention is equivalent among the three groups. PSS = perceived stress scale; * *P*<.05; ** *P*<.001.

**Figure 4:**
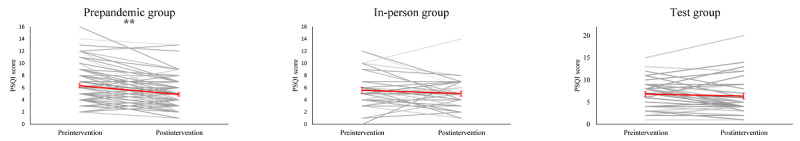
Change in subjective sleep quality. Black lines depict change for individual participants, and red line indicates the mean change and standard errors in the group. Change in self-reported sleep quality was significant in the control group (pre-pandemic) but in neither of the groups trained during the pandemic. PSQI = Pittsburgh Sleep Quality Index; * *P*<.001.

The one-way ANOVA test showed that there were no significant differences in the three groups’ baseline, self-reported sleep quality levels (*F*_2,159_=1.53; *P*=.22). Participants’ PSQI scores were above threshold (ie, a global PSQI score of >5). This suggested that all three groups experienced sleep difficulties. After comparing the effects of the interventions for each group, we found that training had a significant overall effect (*F*_1,155_=4.45; *P*=.04; partial ε^2^=.028). However, there were no interactions between the training and group factors (*F*_2,155_=2.00; *P*=.14) (Figure 3). Our planned posthoc comparisons showed a significant improvement in the prepandemic training group’s sleep quality (*t*_85_=5.37; *P*<.001), but there were no significant changes in PSQI scores between the two groups that received training during the pandemic (in-person training group: *t*_35_=1.16; *P*=.25; test group: *t*_37_=0.96; *P*=.34) (Figure 4).

The two one-tailed *t* tests for comparing changes in PSQI scores between the test group and the prepandemic training group (the smallest effect size of interest was set to 0.29) revealed that the observed effect size was not significantly within the equivalence bounds (*t*_123_=0.93; *P*=.18). This suggested that web-based mindfulness training during the pandemic was not equivalent to in-person training before the pandemic in terms of improving sleep quality. In contrast, the comparison between the test group and the in-person training group (the smallest effect size of interest was set to 0.38) indicated that web-based training during the pandemic was equivalent to in-person training during the pandemic (*t*_72_=3.10; *P*=.001).

### Secondary Sleep Variables

**Figure 5:**
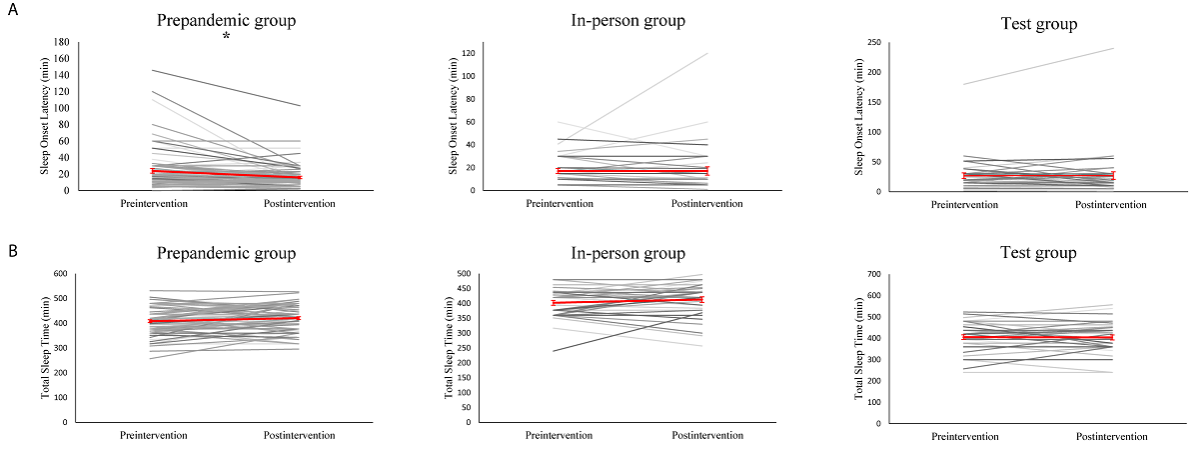
: Changes in sleep variables. Black lines depict change for individual participants, and red line indicates the mean change and standard errors in the group. (A) Sleep onset latency (SOL) decreases significantly on the control group, but not COVID1 or COVID2. (B) No significant changes were observed in total sleep time (TST). * *P*<.001.

We conducted an exploratory analysis on self-reported total sleep times and sleep onset latency. The repeated measures ANCOVA test, which controlled for the effects of course type and previous meditation experience on total sleep time, revealed that there were no significant changes in total sleep time after meditation training (*F*_1,155_=1.76; *P*=.19) and no group by training interactions (*F*_2,157_=0.88; *P*=.42). However, participants in the in-person and prepandemic training groups reported that they experienced 10 more minutes of sleep following mindfulness training (Figure 5). The ANCOVA test for assessing sleep onset latency revealed that training did not have a significant effect (*F*_1,155_=0.44; *P*=.50) and that there were significant group by training interactions (*F*_2,155_=3.80; *P*=.03). Our posthoc comparisons indicated that this interaction was driven by the significant reduction in sleep onset latency in the prepandemic training group (pretraining: mean 23.64 minutes, SD 25.61 minutes; posttraining: mean 15.728 minutes, SD 14.30 minutes; *t*_85_=3.92; *P*<.001) instead of those in the in-person training and test groups (Figure 5).

Our sleep onset latency data contained several outlier values (ie, SDs of >3 from the mean) that were plausible, which may have been the reason for our significant results. Therefore, we reanalyzed the data via nonparametric bootstrap resampling. This involved 5000 reshuffles of the control and test labels [27]. After using this method, we found that training had a significant effect in the prepandemic training group (paired mean difference=−7.92; 95% CI −12.9 to −4.81; *P*<.001). However, training did not have a significant effect in the in-person training group (paired mean difference=0.028; 95 %CI −3.67 to 8.67; *P*=.99) and the test group (paired mean difference=0.134; 95% CI −4.48 to 6.34; *P*=.96). This supported our hypothesis that mindfulness training before the pandemic shortens sleep onset latency and mindfulness training during the pandemic does not (Multimedia Appendix 2).

### Course Attendance and Practice Time

We analyzed the percentage of training sessions that participants attended to account for the different course durations. The two one-tailed *t* tests revealed that the attendance rate of the test group was equivalent to those of the prepandemic training group (*t*_124_=1.86; *P*=.03) and the in-person training group (*t*_124_=2.15; *P*=.02). Course attendance was high in all three groups (prepandemic training group: mean 96.2%, SD 15.9%; in-person training group: 96.5%, SD 8.8%; test group: 96.4%, SD 14.1%).

As the recommended amount of home practice differed between the 4-week and 8-week courses, we only performed a subgroup analysis on the daily practice times of participants in the 4-week courses (prepandemic training group: n=73; in-person training group: n=36; test group: n=27). Participants in the test group had higher daily practice times (mean 15.7 minutes, SD 8.78 minutes) than the prepandemic training group (mean 12.78 minutes, SD 6.19 minutes) and the in-person training group (mean 13.97, SD 8.30 minutes). Our equivalence tests showed that the observed effect size between the test group and the prepandemic training group was not significantly within the equivalence bounds (*t*_120_=−0.83; *P*=.20); however, the effect size between the two groups was not significantly different (*t*_97_=−1.21; *P*=.23). The test group’s and in-person training group’s practice times were statistically equivalent (*t*_61_=−2.19; *P*=.01).

## Discussion

### Principal Findings

Unlike other studies [28,29], we found no evidence of heightened stress and poorer sleep quality (ie, compared to baseline levels) during the COVID-19 pandemic compared to those before the pandemic. These findings may be specific to our participants, who were generally well educated and of relatively high socioeconomic status. Our results are in line with data from a study that had a large sample of working professionals in Singapore. These working professionals exhibited increases in weekend sleep duration and comparable levels of sleep efficiency during the lockdown period compared to those before the lockdown period [30]. Regardless, our participants still reported moderate levels of stress and sleep disturbance on average, suggesting that interventions for improving these outcomes are valuable.

The key finding from this study was that the effects of mindfulness training during the COVID-19 pandemic were equivalent to those of in-person mindfulness training during and before the pandemic in terms of reducing stress. However, we did not find evidence that this training improved sleep quality. These findings remained consistent regardless of whether the training was conducted in person or via a web-based platform. Videoconferencing has emerged as a very useful tool for mental health providers, as it can be used when restrictions have necessitated measures such as social distancing and quarantine. Furthermore, our data support the effectiveness of mindfulness training that is delivered in this format.

To date, there have been little data on the effects of mindfulness training during the COVID-19 pandemic. Although a randomized study that was conducted in Wuhan during the pandemic has reported on the sleep-related benefits of brief mindfulness practices (ie, compared to a mind-wandering control condition) [31], the measures it used were not validated, and the intervention (ie, self-guided practice for 10 minutes per day) was unfacilitated and of relatively low intensity.

A possible reason for the discrepancy between the two outcome variables in this study is that the effect size of training for improving sleep was smaller than that for improving stress, and our sample size was not sufficiently powered to detect this difference. However, the reduction in sleep onset latency in the prepandemic control group was large, and almost no changes in this variable were observed after mindfulness training during the pandemic (Multimedia Appendix 1). This is evidence against our explanation. We thus posit that there are undiscovered factors that relate to coping with a global crisis and specifically weaken the effects of mindfulness training on sleep quality. For example, irregular schedules, less light exposure, and reduced physical activity during a pandemic may play a large role in influencing sleep, and these factors are not targeted by mindfulness training [32]. If this is the case, our data have implications for prescribing mindfulness interventions during a crisis. Our data suggest that mindfulness training participants’ primary desired goal should be stress reduction instead of sleep improvement.

When mindfulness was first introduced in Western medicine, mindfulness instruction was typically delivered in groups, as inquiry and discussion were integral parts of training sessions. With the increasing penetration of technology in society, there has been growing interest in the digital delivery of this training due to its potential for reaching a large number of people at a relatively low cost. Studies on digital mindfulness training have used a variety of dissemination methods; web-based and email-based delivery are the most common [33]. In contrast, relatively few trials have used group videoconferencing, which confers the advantage of allowing both teachers and participants to interact remotely for group sharing and inquiry activities. These are key components that are present in face-to-face training. The need for such high-quality facilitation [34] is an often-ignored aspect in the field of mindfulness research. Digital platforms that do not provide live instruction and allow participants to have real-time discussions with an experienced teacher (ie, a person who embodies the qualities of mindfulness) may result in misunderstandings (ie, with regard to how mindfulness should be practiced) or adverse impacts on participants. Foundational attitudes that are fundamental in mindfulness practice (eg, nonstriving and letting go) may not be intuitive to people who are accustomed to the teleological foundations of treatment in Western medicine. Therefore, in-person guidance and inquiry are particularly important for inexperienced practitioners. Furthermore, videoconferencing preserves the other social elements (eg, peer-to-peer sharing of experiences through the use of Zoom breakout rooms) of the intervention that may be critical to behavioral change.

Videoconferencing as a means of delivering mindfulness training has been tested in a small number of studies, and it generally results in superior outcomes compared to those of untreated controls. However, none of these studies have formally tested the equivalence between videoconferencing-based mindfulness training and in-person mindfulness training, as established by our analysis. For example, Zernicke et al [35] reported moderate reductions in stress and mood disturbance in a randomized, wait-list controlled trial of cancer survivors who participated in a videoconferencing-based, mindfulness-based cancer recovery program. Furthermore, Gardner-Nix et al [36] reported that the positive effects that in-person and remote mindfulness-based pain management have on mental health and pain catastrophizing were superior to those of a wait-list control. However, they did not establish the equivalence of the mindfulness conditions. Beyond fully facilitated treatments, other studies that report on the positive results of web-based training have used individualized mentoring and coaching as an adjunct to self-administered content [37]. Therefore, recent evidence indicates that remotely delivered mindfulness training has beneficial effects on a wide spectrum of different health outcomes.

With regard to treatment acceptance, we found that adherence to the intervention was good; participants attended 96.3% (709/736) of the classes and practiced for an average of 15 minutes per day, as prescribed. This differs from app-based mindfulness training, in which app use tends to decrease over time [38]. This is partially due to difficulties in incorporating app use into a routine [39]. A particular strength of our study protocol was that the mindfulness interventions involved standardized curricula, which were delivered by highly experienced instructors. These interventions have also been tested and reported on in prior studies [15].

### Limitations

This study has a number of limitations that are worth noting. As this was a retrospective analysis, participants in this study were not randomized by condition, thereby introducing the risk of bias (eg, selection bias whereby participants with COVID-19 were more motivated to engage in mindfulness training than those without COVID-19), However, due to the nature of our study, randomization for investigating the effects of training during a crisis period was not possible. Furthermore, we were not able to conduct a priori power analysis, and our sample size was determined by the maximum recruitment of participants within the two target periods. As such, we had to use relatively wide equivalence bounds to test our hypotheses.

We also noted that the postintervention rates of attrition were substantial and imbalanced between groups, and we were unable to rule out the possibility that these dropouts were not random. Regardless, this level of attrition is typical for web-based survey studies with limited experimenter-participant contact [40,41].

Of note, our sample was predominantly Han Chinese and well educated. Further research is needed to determine the generalizability of our reported effects to other populations.

### Future Directions

The findings in this analysis are encouraging. They suggest that prospective randomized controlled trials should be conducted to provide stronger evidence for the effectiveness of videoconferencing. Future studies should also focus on understanding why mindfulness interventions are not effective in terms of improving sleep quality during times of crisis and determining how standard treatments might be adapted to target sleep difficulties.

### Conclusions

Our data suggest that group mindfulness training that is delivered via videoconferencing is as effective as traditional, in-person training in terms of reducing stress. However, in-person and web-based mindfulness training during the pandemic were not comparable to prepandemic mindfulness training in terms of improving sleep quality. Videoconferencing may be an attractive, alternative method of delivering mindfulness training for reducing stress when restrictions make in-person training less viable.

## Appendix

Multimedia Appendix 1 Detailed information of mindfulness training courses.

Multimedia Appendix 2 Estimation plots showing the mean difference and 95% confidence interval between sleep onset latency before and after the mindfulness interventions. 5,000 bootstrap resamples were used to create each distribution. Sleep onset latency decreased significantly in the control group (paired mean difference = -7.92 [95.0% CI -12.9, -4.81], P<.001), but not in COVID1 (paired mean difference = 0.028 [95.0% CI -3.67, 8.67], P=.99) or COVID2 (paired mean difference = 0.134 [95.0% CI -4.48, 6.34], P=.96).

## Abbreviations

ANCOVA: analysis of covariance
ANOVA: analysis of variance
PSQI: Pittsburgh Sleep Quality Inventory
PSS: Perceived Stress Scale

## References

[1] Wang C, Pan R, Wan X, Tan Y, Xu L, McIntyre RS, Choo FN, Tran B, Ho R, Sharma VK, Ho C (2020). A longitudinal study on the mental health of general population during the COVID-19 epidemic in China. Brain Behav Immun.

[2] Tan W, Hao F, McIntyre RS, Jiang L, Jiang X, Zhang L, Zhao X, Zou Y, Hu Y, Luo X, Zhang Z, Lai A, Ho R, Tran B, Ho C, Tam W (2020). Is returning to work during the COVID-19 pandemic stressful? A study on immediate mental health status and psychoneuroimmunity prevention measures of Chinese workforce. Brain Behav Immun.

[3] Hao F, Tan W, Jiang L, Zhang L, Zhao X, Zou Y, Hu Y, Luo X, Jiang X, McIntyre RS, Tran B, Sun J, Zhang Z, Ho R, Ho C, Tam W (2020). Do psychiatric patients experience more psychiatric symptoms during COVID-19 pandemic and lockdown? A case-control study with service and research implications for immunopsychiatry. Brain Behav Immun.

[4] Ye Z, Yang X, Zeng C, Wang Y, Shen Z, Li X, Lin D (2020). Resilience, social support, and coping as mediators between COVID-19-related stressful experiences and acute stress disorder among college students in China. Appl Psychol Health Well Being.

[5] Ho CS, Chee CY, Ho RC (2020). Mental health strategies to combat the psychological impact of COVID-19 beyond paranoia and panic. Ann Acad Med Singap.

[6] Kabat-Zinn J (1994). Wherever You Go, There You Are: Mindfulness Meditation for Everyday Life.

[7] Kabat-Zinn J (1991). Full Catastrophe Living: Using the Wisdom of Your Body and Mind to Face Stress, Pain, and Illness.

[8] Khoury B, Sharma M, Rush SE, Fournier C (2015). Mindfulness-based stress reduction for healthy individuals: A meta-analysis. J Psychosom Res.

[9] Khoury B, Lecomte T, Fortin G, Masse M, Therien P, Bouchard V, Chapleau MA, Paquin K, Hofmann SG (2013). Mindfulness-based therapy: a comprehensive meta-analysis. Clin Psychol Rev.

[10] Creswell JD, Pacilio LE, Lindsay EK, Brown KW (2014). Brief mindfulness meditation training alters psychological and neuroendocrine responses to social evaluative stress. Psychoneuroendocrinology.

[11] Britton WB, Shahar B, Szepsenwol O, Jacobs WJ (2012). Mindfulness-based cognitive therapy improves emotional reactivity to social stress: results from a randomized controlled trial. Behav Ther.

[12] Kober H, Brewer JA, Height KL, Sinha R (2017). Neural stress reactivity relates to smoking outcomes and differentiates between mindfulness and cognitive-behavioral treatments. Neuroimage.

[13] Parmentier FBR, García-Toro M, García-Campayo J, Yañez AM, Andrés P, Gili M (2019). Mindfulness and symptoms of depression and anxiety in the general population: The mediating roles of worry, rumination, reappraisal and suppression. Front Psychol.

[14] Howell AJ, Digdon NL, Buro K (2010). Mindfulness predicts sleep-related self-regulation and well-being. Pers Individ Dif.

[15] Hassirim Z, Lim ECJ, Lo JC, Lim J (2019). Pre-sleep cognitive arousal decreases following a 4-week introductory mindfulness course. Mindfulness (N Y).

[16] Ong JC, Manber R, Segal Z, Xia Y, Shapiro S, Wyatt JK (2014). A randomized controlled trial of mindfulness meditation for chronic insomnia. Sleep.

[17] Chattu VK, Manzar MD, Kumary S, Burman D, Spence DW, Pandi-Perumal SR (2018). The global problem of insufficient sleep and its serious public health implications. Healthcare (Basel).

[18] Home - Brahm Centre. Brahm Centre.

[19] Carmody J, Baer RA (2008). Relationships between mindfulness practice and levels of mindfulness, medical and psychological symptoms and well-being in a mindfulness-based stress reduction program. J Behav Med.

[20] Strohmaier S (2020). The relationship between doses of mindfulness-based programs and depression, anxiety, stress, and mindfulness: a dose-response meta-regression of randomized controlled trials. Mindfulness (N Y).

[21] SurveyMonkey: The world's most popular free online survey tool. SurveyMonkey.

[22] Video conferencing, web conferencing, webinars, screen sharing - Zoom. Zoom.

[23] Cohen S, Kamarck T, Mermelstein R (1983). A global measure of perceived stress. J Health Soc Behav.

[24] Buysse DJ, Reynolds 3rd CF, Monk TH, Berman SR, Kupfer DJ (1989). The Pittsburgh Sleep Quality Index: a new instrument for psychiatric practice and research. Psychiatry Res.

[25] Lakens D (2017). Equivalence tests: A practical primer for t tests, correlations, and meta-analyses. Soc Psychol Personal Sci.

[26] Julian Lim. Open Science Framework.

[27] Ho J, Tumkaya T, Aryal S, Choi H, Claridge-Chang A (2019). Moving beyond *P* values: data analysis with estimation graphics. Nat Methods.

[28] Wang C, Pan R, Wan X, Tan Y, Xu L, Ho CS, Ho RC (2020). Immediate psychological responses and associated factors during the initial stage of the 2019 coronavirus disease (COVID-19) epidemic among the general population in China. Int J Environ Res Public Health.

[29] Flesia Luca, Monaro Merylin, Mazza Cristina, Fietta Valentina, Colicino Elena, Segatto Barbara, Roma Paolo (2020). Predicting Perceived Stress Related to the Covid-19 Outbreak through Stable Psychological Traits and Machine Learning Models. J Clin Med.

[30] Ong JL, Lau T, Massar SAA, Chong ZT, Ng BKL, Koek D, Zhao W, Yeo BTT, Cheong K, Chee MWL (2021). COVID-19-related mobility reduction: heterogenous effects on sleep and physical activity rhythms. Sleep.

[31] Zheng M, Yao J, Narayanan J Mindfulness buffers the impact of COVID-19 outbreak information on sleep duration. PsyArXiv..

[32] Appleman K, Figueiro MG, Rea MS (2013). Controlling light-dark exposure patterns rather than sleep schedules determines circadian phase. Sleep Med.

[33] Russell L, Ugalde A, Milne D, Austin D, Livingston PM (2018). Digital characteristics and dissemination indicators to optimize delivery of internet-supported mindfulness-based interventions for people with a chronic condition: Systematic review. JMIR Ment Health.

[34] Crane RS, Kuyken W, Williams JMG, Hastings RP, Cooper L, Fennell MJV (2012). Competence in teaching mindfulness-based courses: Concepts, development and assessment. Mindfulness (N Y).

[35] Zernicke KA, Campbell TS, Speca M, McCabe-Ruff K, Flowers S, Carlson LE (2014). A randomized wait-list controlled trial of feasibility and efficacy of an online mindfulness-based cancer recovery program: the eTherapy for cancer applying mindfulness trial. Psychosom Med.

[36] Gardner-Nix J, Backman S, Barbati J, Grummitt J (2008). Evaluating distance education of a mindfulness-based meditation programme for chronic pain management. J Telemed Telecare.

[37] Segal ZV, Dimidjian S, Beck A, Boggs JM, Vanderkruik R, Metcalf CA, Gallop R, Felder JN, Levy J (2020). Outcomes of online mindfulness-based cognitive therapy for patients with residual depressive Symptoms: A randomized clinical trial. JAMA Psychiatry.

[38] Flett JAM, Hayne H, Riordan BC, Thompson LM, Conner TS (2018). Mobile mindfulness meditation: a randomised controlled trial of the effect of two popular apps on mental health. Mindfulness (N Y).

[39] Laurie J, Blandford A (2016). Making time for mindfulness. Int J Med Inform.

[40] Daikeler J, Bošnjak M, Manfreda KL (2019). Web versus other survey modes: An updated and extended meta-analysis comparing response rates. J Surv Stat Methodol.

[41] Sánchez-Fernández J, Muñoz-Leiva F, Montoro-Ríos FJ (2012). Improving retention rate and response quality in web-based surveys. Comput Human Behav.

