# Supplementary material for: Effects of Web-Based Group Mindfulness Training on Stress and Sleep Quality in Singapore During the COVID-19 Pandemic: Retrospective Equivalence Analysis
Source: JMIR Ment Health. 2021 Mar 15;8(3):e21757. doi: 10.2196/21757 (PMC7962857; doi:10.2196/21757)
Supplement: Multimedia Appendix 1 [file mental_v8i3e21757_app1.docx]

**Supplementary Information**

**Details of mindfulness training courses**

Participants completed one of three mindfulness courses offered by Brahm Centre; the Mindfulness Foundation Course, the Mindfulness Intermediate Course, or the Mindfulness-Based Stress Reduction Course. All courses were conducted by experienced Brahm Centre instructors. All lessons consisted of didactic teaching, group discussion, inquiry, and formal mindfulness practices. Participants were provided with weekly session handouts and guided audio meditation. They were encouraged to practice informal mindfulness practices of mindful eating, mindful walking and mindfulness of daily activities. There was no explicit focus on sleep education or improving sleep quality in all of the courses.

**Mindfulness Foundation Course**

The Mindfulness Foundation Course is a weekly 2-hour, 4-session group-based mindfulness intervention designed by Brahm Centre. Participants engaged in 10-15 min of formal mindfulness practices including body scan, breath awareness, loving-kindness meditation.

The themes for the 4 sessions were “Introduction to Mindfulness,” “Be Right Where You Are,” “Our Storytelling Minds,” and “Cultivating Kindness.” In the first session, participants were introduced to mindfulness and explored 6 foundational attitudes of mindfulness practice. In the second session, participants explored the habits of the mind, identified their stress triggers and learnt the value of being present with awareness. The third session invited participants to investigate the power of their storytelling minds and how it may have affected their stress levels. The final session ended with an invitation to work with difficult emotions and thoughts through cultivation of kindness towards oneself and others.

**Mindfulness Intermediate Course**

The Mindfulness Intermediate Course is a weekly 2-hour, 4-session group-based mindfulness intervention designed by Brahm Centre. The course is targeted at participants who completed the Mindfulness Foundation Course, empowering them to deepen and build on what was learnt previously. Participants engaged in daily mindfulness practices including mindful walking, affectionate body scan and compassionate breathing, lasting 20-25 min for each practice.

The themes for the 4 sessions were “Willingness to Be with Things as They Are”, Finding Compassion Within,” “Enhancing Resilience” and “Moving Forward in Mindfulness.” In the first session, participants revisited the foundational attitudes taught in the Mindfulness Foundation Course, with the focus on working with resistance and exploring choices. The second session introduced the concept of self-compassion and ways to develop greater equanimity and kindness towards self and others. The third session offered participants different strategies to enhance resilience and overcome adversity. The last session reinforced participants’ commitment to mindfulness practice by offering a myriad of ways to weave mindfulness throughout their daily lives.

**Mindfulness-Based Stress Reduction Course**

The Mindfulness-Based Stress Reduction Course is a weekly 2.5-hour, 8-session group-based mindfulness intervention, including a 7.5-hour ‘all-day retreat’. This course was developed by Jon Kabat-Zinn in 1979. Participants engaged in formal mindfulness practices including body scan, awareness of breath, standing/lying-down yoga, sitting meditation and loving-kindness meditation. They were given daily mindfulness practices lasting 45 mins to complete in between classes.

The themes of the 8 session were “Introduction to Mindfulness,” “Perception Matters,” “Being Present,” “Stress Reactivity,” “Stress Response,” “Interpersonal Mindfulness,” “Applying Mindfulness in Daily Life” and “Maintaining Momentum in Mindfulness”.

For online administrations of these courses through Zoom video-conferencing, the retreat was omitted from the curriculum.

**Supplementary Table 1.** Participant breakdown by individual courses

| **Group** | **Course dates** | **Course type** | **Format** | **Class size** | **Number enrolled in study** |
| --- | --- | --- | --- | --- | --- |
| CTRL | 3/10/19 to 21/11/19 | MBSR | In-person | 32 | 13 |
| CTRL | 15/10/19 to 5/11/19 | MFC | In-person | 34 | 11 |
| CTRL | 5/11/19 to 26/11/19 | MFC | In-person | 70 | 19 |
| CTRL | 6/11/19 to 27/11/19 | MFC | In-person | 45 | 21 |
| CTRL | 28/11/19 to 19/12/19 | MFC | In-person | 76 | 22 |
| COVID1 | 1/2/20 to 22/2/20 | MFC | In-person | 79 | 17 |
| COVID1 | 5/2/20 to 26/2/20 | MFC | In-person | 25 | 6 |
| COVID2 | 3/3/20 to 21/4/20 | MBSR | Online | 21 | 6 |
| COVID1 | 5/3/20 to 26/3/20 | MFC | Online | 37 | 13 |
| COVID2 | 18/3/20 to 6/5/20 | MBSR | Online | 15 | 5 |
| COVID2 | 20/3/20 to 10/4/20 | MIC | Online | 11 | 3 |
| COVID2 | 2/4/20 to 23/4/20 | MFC | Online | 32 | 11 |
| COVID2 | 8/4/20 to 29/4/20 | MFC | Online | 20 | 4 |
| COVID2 | 5/5/20 to 26/5/20 | MFC | Online | 14 | 4 |
| COVID2 | 8/5/20 to 29/5/20 | MFC | Online | 12 | 5 |

MFC = Mindfulness Foundation Course; MIC = Mindfulness Intermediate Course; MBSR = Mindfulness-Based Stress Reduction
