# Supplementary figures and images for: Effects of Web-Based Group Mindfulness Training on Stress and Sleep Quality in Singapore During the COVID-19 Pandemic: Retrospective Equivalence Analysis
Source: JMIR Ment Health. 2021 Mar 15;8(3):e21757. doi: 10.2196/21757 (PMC7962857; doi:10.2196/21757)

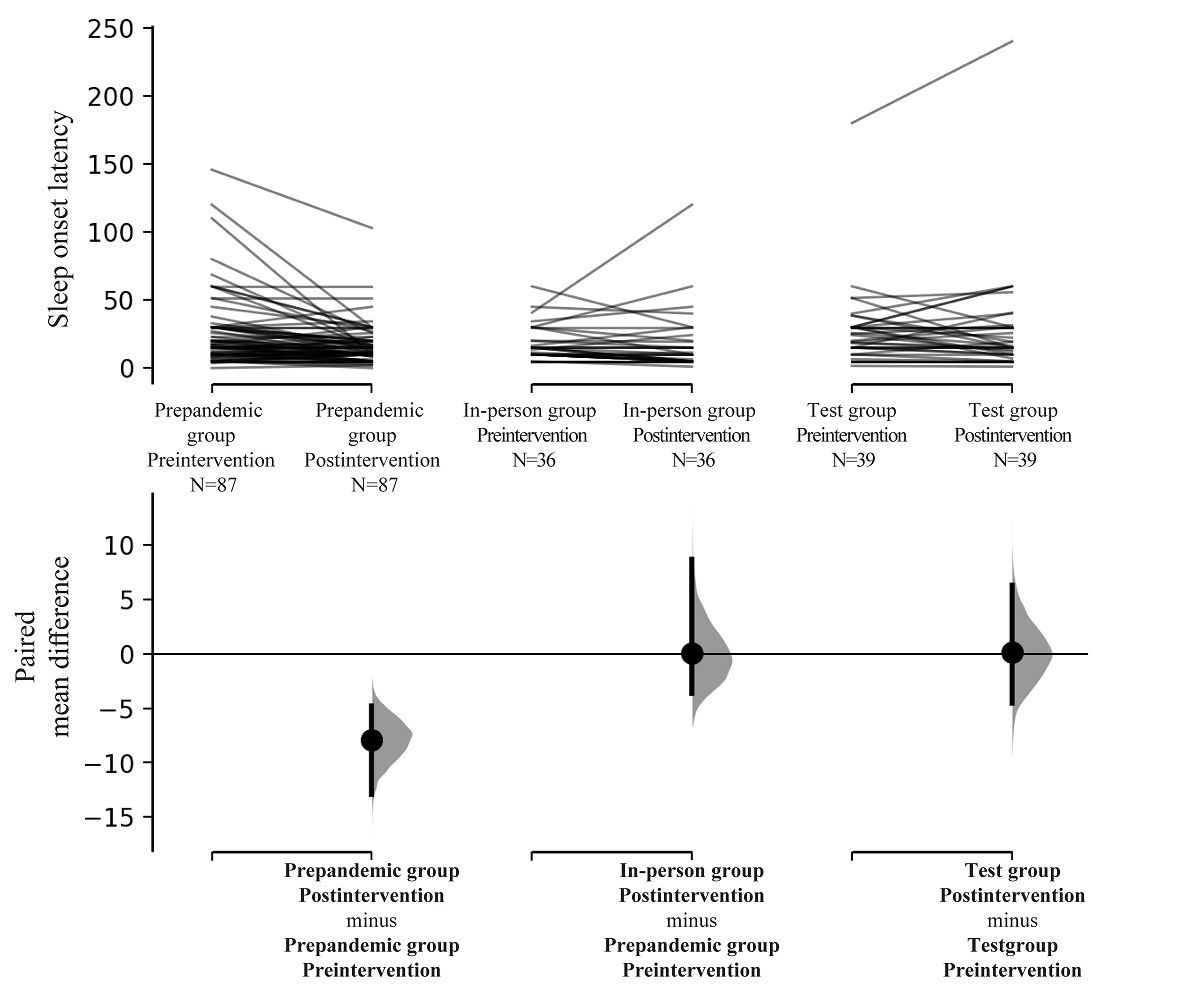

Supplement: Multimedia Appendix 2 [file mental_v8i3e21757_app2.png]
